# Utilizing Descriptive Statements from the Biodiversity Heritage Library to Expand the Hymenoptera Anatomy Ontology

**DOI:** 10.1371/journal.pone.0055674

**Published:** 2013-02-18

**Authors:** Katja C. Seltmann, Zsolt Pénzes, Matthew J. Yoder, Matthew A. Bertone, Andrew R. Deans

**Affiliations:** 1 Department of Invertebrate Zoology, American Museum of Natural History, New York, New York, United States of America; 2 Department of Ecology, University of Szeged, Szeged, Csongrád, Hungary; 3 Species File, Prairie Research Institute, Champaign, Illinois, United States of America; 4 Department of Entomology, North Carolina State University, Raleigh, North Carolina, United States of America; 5 Department of Entomology, Pennsylvania State University, University Park, Pennsylvania, United States of America; Field Museum of Natural History, United States of America

## Abstract

Hymenoptera, the insect order that includes sawflies, bees, wasps, and ants, exhibits an incredible diversity of phenotypes, with over 145,000 species described in a corpus of textual knowledge since Carolus Linnaeus. In the absence of specialized training, often spanning decades, however, these articles can be challenging to decipher. Much of the vocabulary is domain-specific (e.g., Hymenoptera biology), historically without a comprehensive glossary, and contains much homonymous and synonymous terminology. The Hymenoptera Anatomy Ontology was developed to surmount this challenge and to aid future communication related to hymenopteran anatomy, as well as provide support for domain experts so they may actively benefit from the anatomy ontology development. As part of HAO development, an active learning, dictionary-based, natural language recognition tool was implemented to facilitate Hymenoptera anatomy term discovery in literature. We present this tool, referred to as the ‘Proofer’, as part of an iterative approach to growing phenotype-relevant ontologies, regardless of domain. The process of ontology development results in a critical mass of terms that is applied as a filter to the source collection of articles in order to reveal term occurrence and biases in natural language species descriptions. Our results indicate that taxonomists use domain-specific terminology that follows taxonomic specialization, particularly at superfamily and family level groupings and that the developed Proofer tool is effective for term discovery, facilitating ontology construction.

## Introduction

The vast majority of our biological knowledge exists only in printed, prosaic natural language, or ‘analog’ texts [1]. This situation is equally true for the field of descriptive taxonomy, the subdomain of biology responsible for describing organisms and classifying them into nested sets cataloged with scientific names (i.e. taxa). Publication protocol for the description of a new animal species requires that an organism ‘diagnosis’ (list of distinguishing characteristics) for each new taxon be published in a journal in accordance with the International Code of Zoological Nomenclature [2] and until 2011 these journals had to be printed in journals with paper copies. Recent modifications in the code now allow for entirely electronic publication under certain conditions [3]. Language usage for these diagnoses is dependent on the describing authors, journal editors, and reviewers of the manuscript, without standardized vocabularies across domains. Analog descriptions about our domain (Hymenoptera) posed a challenge for development of the Hymenoptera Anatomy Ontology (HAO) [4] as well as other anatomy ontology projects, which aim, in part, is to capture lexica from legacy literature. The primary goal of this effort was to propose a method of efficiently surveying the literature for terms (definition of term sensu Seltmann et al., 2012) [5] and in the process observe trends by analyzing term occurrence in species descriptions. The collected anatomical (i.e. morphological) terms were applied to the construction of the HAO, based on principles of structural similarity [5], [6] enabling future diagnoses to be tied a priori to a structured vocabulary that is detailed enough in morphological terminology to be effective for comparable and accurate descriptions [7], [8].

## Materials and Methods

### Term Collection

In the biological sciences one of the important and growing online resources is the Biodiversity Heritage Library (BHL) [9], a clearinghouse for legacy literature, all of which is scanned and subsequently optically character recognized (OCRed). The International Society of Hymenoptera (ISH) [10] archives its *Journal of Hymenoptera Research* (*JHR*) in the BHL. We extracted OCR text for JHR (1993–2007, the latest year available at the time of data collection) from the BHL and manually partitioned the 353 articles for upload into the mx database [11]. Mx is a Web-based, open source set of tools for descriptive taxonomy with recent advances to support collaborative ontology development. When this exercise was conducted the BHL Application Programming Interface (API) did not return OCR of specific articles, only of entire issues of the journal. Processing of the BHL OCR required manually cutting and pasting the text into the database. We made no attempt to correct the OCR output. Associated metadata, including reference citation, was associated with each article. Citations were collected using Zotero [12] after Google Scholar [13] searches returned citations in Endnote [14] format, and these citations were then uploaded into the mx database using a custom Endnote importation tool.

**Figure 1:**
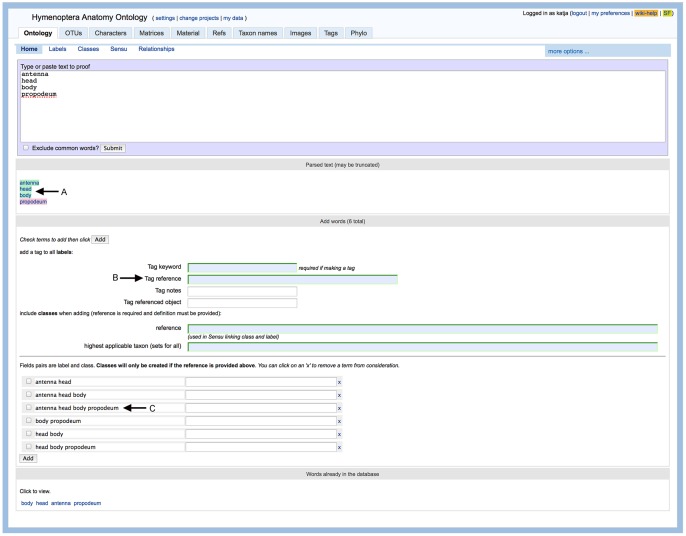
Screenshot of the mx interface for string matching terms in the database with OCR text. Possible additional new terms are proposed for the user to include.

Once the articles were in the database, a simple dictionary-based, entity recognition tool was developed in mx to match terms captured for the HAO within blocks of text. The tool, or ‘Proofer’, uses string matching, allowing for commonly found exceptions and special cases, thus reducing the impact of malformed OCR commonly found in the BHL-delivered JHR text. The Proofer displays for the user a list of matches on terms in the ontology (highlighted and linked to the display page for that term; figure 1-A) but also presents a proposed list of terms that could be added to the database if the user chooses. In order to create this list, sentences are first broken down into phrases by splitting sentences at small words (1–3 characters long), removing those small words, and splitting at punctuation (period, comma, semi-colon, etc). These phrases are then displayed to the user in a list format starting with a single unmatched word, or term not already in the database, and 1–5 flanking words expanded from left to right (figure 1-C). Users then browse the list of proposed unmatched terms and select those that should be added to the database; thus user (human) input is necessary in the final addition of terms to the database. Adding flanking words reveals more complex anatomical labels such as ‘propleural arm muscle’ where ‘propleural arm’ may already be a label in the database but ‘propleural arm muscle’ may not. All terms added in this manner were annotated (‘tagged’) as JHR-BHL entered objects so that future analyses of the terms collected during this exercise was possible (tag field illustrated, figure1-B). Also, in order to reduce the number of potential terms presented to the reviewer, active learning [15] was employed in a feedback mechanism between application and user.

Words presented to the user for possible inclusion into the database that are not selected by the user are added to a stop words table. If a word is rejected by the user 10 times (i.e. from ten separate articles) that word is added to the final stop words list and no longer presented in subsequent articles, thus reducing the total number of words presented to the user for evaluation. Links to the source code for mx (including the Proofer) are available in the Supplementary Material (S1).

### Comparison to Related Text Processing Applications

CharParser [16] and GoldenGATE [17]–[19] are both applications for examining taxonomic descriptions contained in legacy literature. GoldenGATE is an editor for marking up the text of an entire article, and transforming it into an XML structured document following TaxonX schema. It uses sophisticated pattern matching rules along with subsequent human editing to define a document’s structure. Among the elements identified are the general sections of a ‘taxonomic treatment’, external identifiers (LSIDs), and taxon names. At the present time, individual descriptive statement mark-up has not been realized in GoldenGATE, although interest does exist to include character level semantic annotation in the TaxonX schema [20]. CharParser is a semi-automated semantic annotation system, capturing individual descriptive statements in a structured XML document. In order to facilitate annotation, CharParser develops an independent glossary of qualitative and quantitative terms during the text mining and training process. This aspect of the CharParser application is similar to the Proofer, as it is a lexicon builder enhanced by a human user. CharParser, however, attempts to attain not only the term from a publication, but also to discern its inherent meaning. This is analogous to the Proofer tool plus mx database, as terms collected by the Proofer are eventually associated with ontology concepts via later stages of the ontology building process by domain experts.

Other string matching software exists for examining BHL-generated OCR text, primarily focused on taxon name discovery. TaxonFinder [21] and NetiNeti [22] determine relevant BHL articles for a user by utilizing a controlled vocabulary, or taxon name lists. Although anatomy ontology term usage in descriptive articles has potential as a viable method for literature discovery, at present the Proofer only examines articles specifically chosen for evaluation by a user, i.e. those identified as descriptive works in the domain of interest.

### Analysis of Collected Terms

For each of the 353 articles a small amount of metadata (as ‘tags’) was captured in the database to facilitate creating lists of terms specific for analysis. First, the articles were reviewed and placed into one of two categories: ‘description of new taxon’ or ‘non-description’. Articles were deemed descriptive based on the use of the words ‘description of’ in the article title or if taxonomic treatments were contained within the body of the article. Additionally for each article, the name of the taxon being described was captured in the database at the family level. Finally, terms representing morphological (i.e. anatomical) concepts and those representing qualitative concepts were differentiated.

The resulting data were then used to produce text files useful in R [23] (version 2.11.1), creating an occurrence (presence/absence) matrix using anatomical terms as characters and articles as terminals, with each article tied to a taxon as described within the article. Terms designated as characters were limited to morphological terms and totaled 816. Qualitative terminology (i.e. ‘shiny’, ‘brown’, ‘rugulose’) was not included in the dataset. The terms ‘cell’, ‘area’ and ‘costa’ were removed from the character list as these terms are commonly used in other disciplines besides descriptive biology and often had non-morphological meaning in descriptions. 179 articles were used as terminals, representing 35 families and 10 superfamilies.

**Figure 2:**
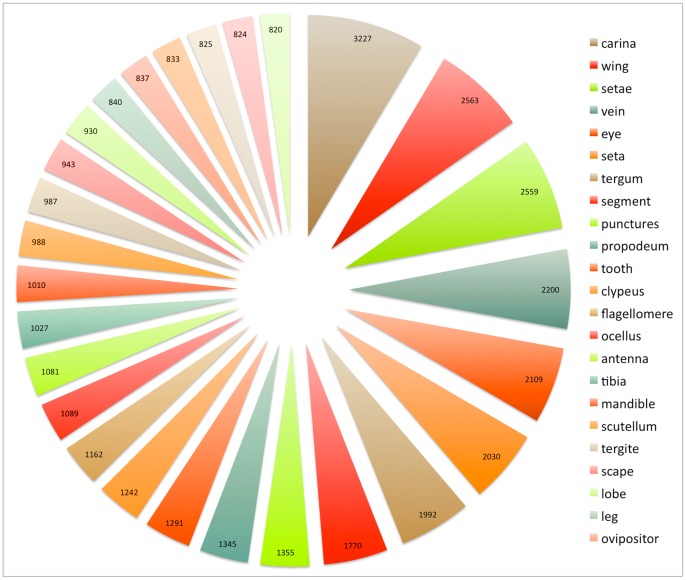
Most commonly used anatomical terms in Hymenoptera. Terms in this figure are ranked based on occurrence among all articles (how many articles a term occurred). Number on chart and size of pie represents the number of total times the term occurred in all articles.

**Figure 3:**
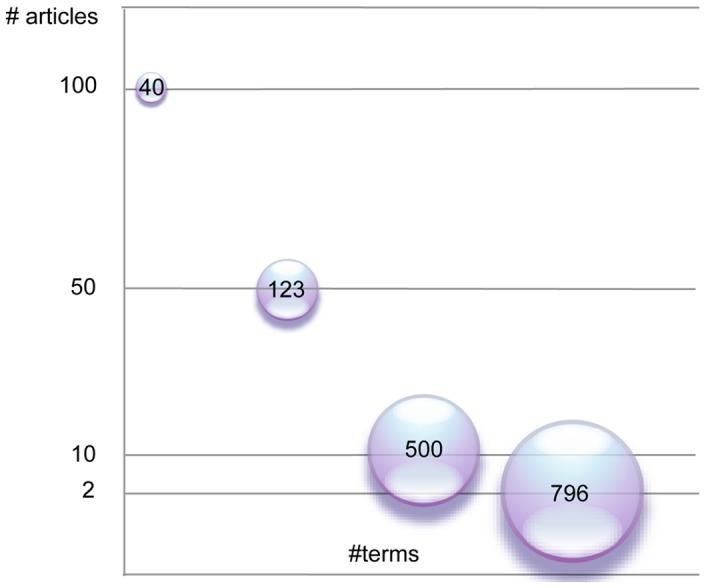
The number of characters (terms) present in at least 2, 10, 50, and 100 articles.

Synonyms and plural terms were summed in the analysis and terms were analyzed as they were recorded in the database. The characters were scored in a binary matrix as presence (1) or absence (0) of the term occurrence within the text of a given article. Four permutations of the matrix were created based on the occurrence of a term. Analyses were preformed that included terms that occurred 2, 10, 50 and 100 times in at least one article. Choosing articles for analysis based solely on occurrence of a term in all articles did not retrieve discrete articles sets at higher numbers, as common terms (figure 2) are ubiquitous. Restricting the terms included in analysis limited the number of included terms to: 796, 500, 123 and 40 respectively (figure 3).

**Figure 4:**
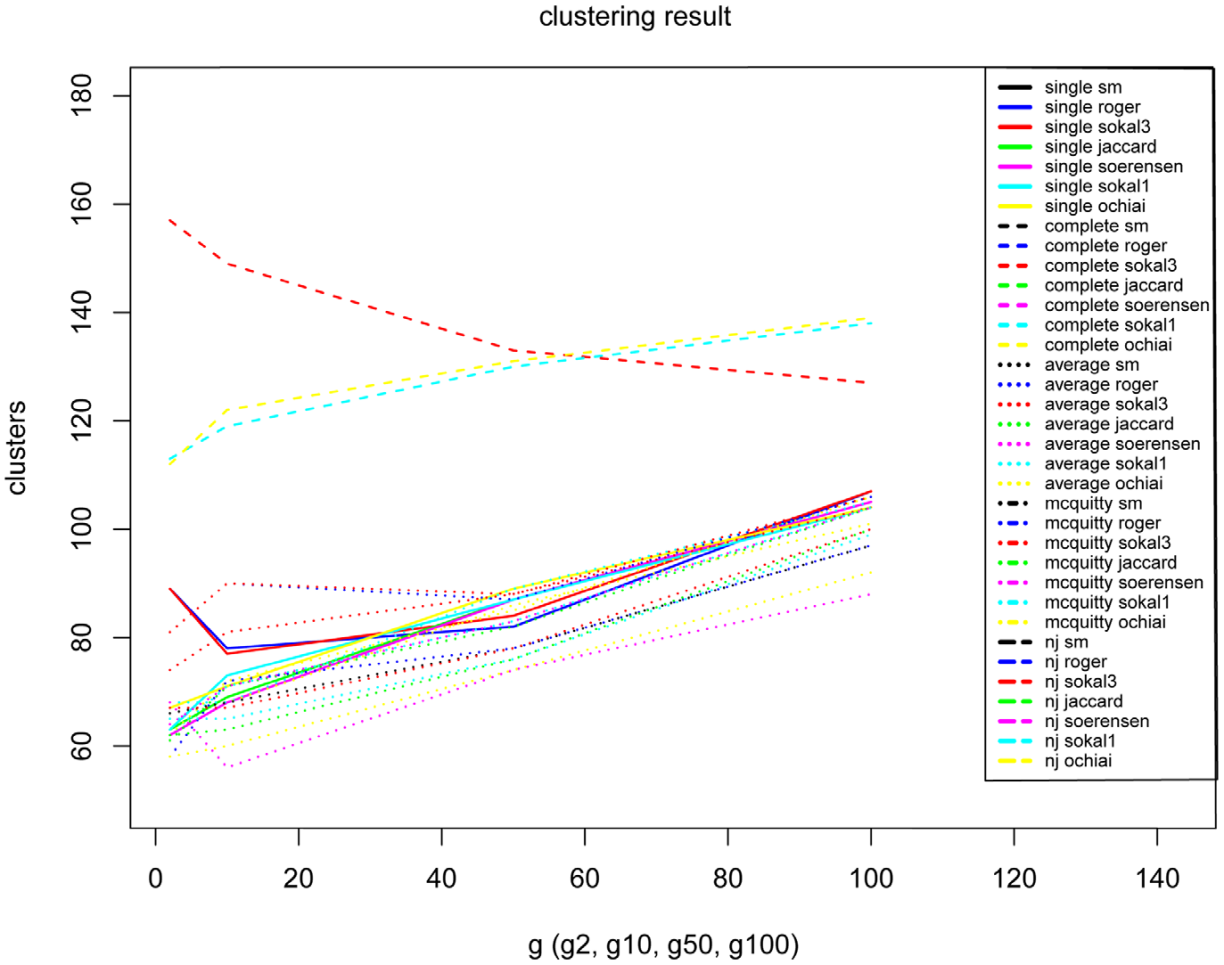
Variation of number of returned clusters based on clustering method and term occurrence in articles.

In order to assess the occurrence of terms contained within articles, matrices were investigated using agglomerative hierarchical clustering methods performed in the R [23] (version 2.11.1) using packages ‘stats’ [23], ‘simba’ [24], ‘vegan’ [25], and ‘ape’ [26]. The range of recovered groups (clusters) on the trees varied from 59–160 based on which analysis method was used (see figure 4 below). Groups were revealed by trimming trees after analysis, and evaluated based on two criteria. First, family and superfamily membership was assigned to each terminal, based on the taxa described in the article. A family or superfamily is a group of organisms based on shared characteristics, associated together under the auspices of the classification hierarchical system, under which other groupings (tribe, subfamily, genus, species) are clustered. Superfamilies are groups that contain multiple families. These families and superfamilies are generally listed in the analyzed journal article; if not, the taxon was placed according to our present understanding of Hymenoptera relationships. Once terminals (taxa) were assigned to a family/superfamily the trees were pruned according to these groups. For example, if two terminals belonged to the same family, and reached the next internal node, they were considered belonging to the same group. Ideally, 10 groups of superfamilies and 37 of families was expected, as this is the number of Hymenoptera families/superfamilies published in the JHR articles used in the analysis.

**Figure 5:**
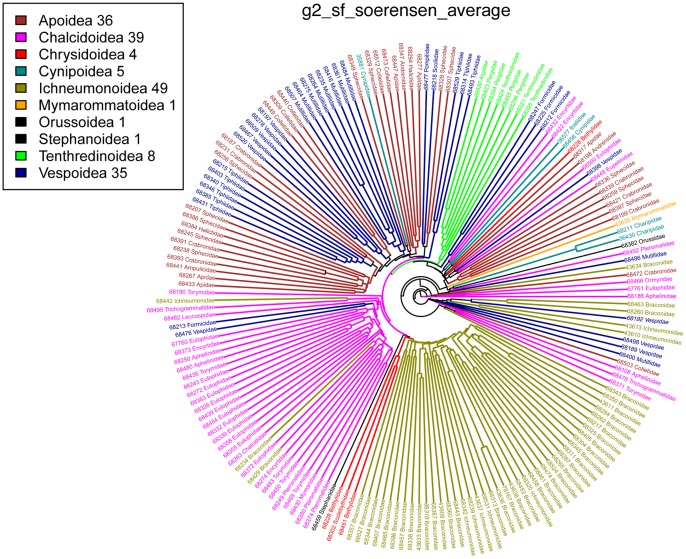
Sorensen Average tree with superfamily name, and number of groupings calculated to superfamily level. The tree represented is the entire, untrimmed tree and the number after the superfamily is the number of groupings retrieved when the tree is trimmed.

The grouping of terminals on the basis of binary characters was extensively investigated by agglomerative hierarchical clustering using the linkage methods (single, complete, average (UPGMA) [27] and McQuitty (WPGMA)) and neighbor-joining (NJ) [28]. Figure 4 outlines the range of outcomes based on which clustering method was chosen. The former result in ultrametric trees (dendrograms, which are ‘rooted’), while the result of the NJ method approaches an additive tree (unrooted) that is based on optimization of the distance on the whole tree [28]. Seven different metric distances were selected, 3 of which were symmetric (incorporate absence matching) and 4 were asymmetric (absence matching is ignored) as follows Kaufman & Rousseeuw, 1990 [29] and Legendre and Legendre 1998 [30]. The Sorensen-Average results tree is included (figure 5) to visually illustrate the grouping results because it most accurately follows groupings expected for Hymenoptera. All other trees and analysis files are available in the supplementary material (S2).

## Results

From the 353 articles we collected 1189 new morphological terms used by Hymenoptera taxonomists. These were added to the mx database, augmenting the development of the Hymenoptera Anatomy Ontology. The Proofer tool developed to assist analysis of these articles improved the efficiency of term extraction from legacy literature by reducing the number of terms presented to the user for review. Comparison of the number of terms presented to the user with and without the Proofer stop words list for 25 randomly selected articles demonstrated that the Proofer stop word list reduced the number of terms displayed to the user by 1/3 of the total actual word count of the article, which was an 80% reduction in the number of combinations of words displayed to a user by the Proofer tool.

180 of the 353 articles were identified to contain descriptions of new taxa, wholly or in part. The most frequently found anatomical terms in those 180 articles are listed in figure 2.

The shortest tree was returned from the Sorensen Average cluster analysis, including characters that were coded for 2 or more terminals, and pruned to superfamily level. This tree results in 63 distinct groupings when the tree was pruned, with observable large clusters of Ichneumonoidea, Chalcidoidea, Symphyta, and Aculeata (figure 5).

## Discussion

The Proofer application and workflow presented here allows for reviewing descriptive text relatively quickly for new terms to supplement the construction of anatomy ontologies. The workflow required the input of domain experts, and open access publications, resulting in the collection of 1189 new terms for the HAO. Although the Proofer tool accumulated numerous terms for inclusion in the HAO, mapping terms to existing classes or creating ontology compliant definitions for those concepts requires further expertise and citation. At present only 144 of the collected terms are tied to HAO concepts. To define concepts, HAO curators initially selected literature that was generally inclusive, taxonomically, of Hymenoptera (including glossary and online resources). This process was done, in part, prior to looking at the BHL JHR articles. Most of the very common terms were already included in the database prior to the term discovery exercise, leaving predominantly highly granular, and superfamily-specific terms used in taxonomic descriptions to be discovered using the Proofer, accounting for the low number of these terms presently fully incorporated in the HAO. These terms will be utilized as the HAO continues to grow, and curators focus on publications from domain experts working exclusively within superfamilies, as this is where the term granularity is demonstrated.

The importance of domain expertise in the process of incorporating these terms cannot be overstated. Jenesen and Bork, 2010 [31] clearly regards input from biologists as necessary for success in biomedical, ontology based literature mining and Dahdul et al. [32] described the importance of taxon experts for phenotype annotation curation. Our evaluation concurs with their observation and extends the thought to conclude that granular terminology is necessary to capture morphological variation, but it requires domain expertise, and evaluation of their publications, in the process, to identify these terms and fully utilize them in the ontology.

Cluster analysis lends evidence for the observation that the Hymenoptera community tends to use granular, domain-specific (i.e., taxon-specific) terminology. Datasets were analyzed extensively using different permutations of clustering methods and datasets delimited by term occurrence. As expected, a high amount of variation in the number of groups recovered was observed in the analysis results. Not all hymenopteran families were in analysis, because taxonomic descriptions of some groups were not published in *JHR* between 1993–2007. Also, there is a strong bias in the number of papers concerning Ichneumonoidea and Chalcidoidea represented. This is due, in part, to the large number of taxonomists interested in these diverse superfamilies. Despite these idiosyncrasies in the data obvious groupings for Ichneumonoidea, Chalcidoidea, “Symphyta” and Aculeata were retrieved. On a more detailed level, many family level groupings were recovered, demonstrating that we can group articles, and those taxa described in the articles, simply by the terms used to describe those organisms. In the comparison of cluster analysis, the number of clusters recovered decreased with an increase of characters (terms) used in the analysis. The terms found more commonly are generally used across Hymenoptera. Terms like head, wing, and carina are almost universally used, and thus provide very little signal to group articles. In order to capture the variation in the terminology of the authors, to manifest any observable signal in the analysis, much less frequently used terms needed to be included.

The corpus of biological literature will continue to grow and with it the need for more automated methods to utilize and discover the information contained within the articles. Natural language processing methods for biological data discovery is only possible through open access publications, and efforts such as the Biodiversity Heritage Library to make legacy literature freely available. This exercise to observe trends in the terminology illustrates how the accessibility to literature facilitates anatomy ontology construction, and an underlying community trend toward domain specificity and, thus, disparate term usage, one of the primary justifications for unifying Hymenoptera terminology through the HAO.

## Supporting Information

S1 Supplementary Material The most recent version of mx code, including the Proofer tool, is available through SourceForge (http://purl.oclc.org/NET/mx-database). The specific version of mx used during analysis is archived on SourceForge and in this combined file.
(ZIP)

S2 Supplementary Material The specific JHR BHL article list, list of terms present in the mx database, and R-scripts used in analysis are supplied in this combined file. These files, and all resulting trees, are additionally archived in the Dryad data repository (doi:10.5061/dryad.3g57k).
(ZIP)
