# Supplementary material for: Utilizing Descriptive Statements from the Biodiversity Heritage Library to Expand the Hymenoptera Anatomy Ontology
Source: PLoS One. 2013 Feb 18;8(2):e55674. doi: 10.1371/journal.pone.0055674 (PMC3575469; doi:10.1371/journal.pone.0055674)
Supplement: S1 Supplementary Material — The most recent version of mx code, including the Proofer tool, is available through SourceForge (http://purl.oclc.org/NET/mx-database). The specific version of mx used during analysis is archived on SourceForge and in this combined file. (ZIP) [file pone.0055674.s001.zip › ref_edge/app/views/ce/labels_print_preview.html.erb]

MX - labels 


<% col = 1; lpc = 0 -%>

<% @labels.each do |l| -%>
<% cllc = (l.print\_label.blank? ? 0 : l.print\_label.count("\n")) -%>
<% for c in 0..l.num\_to\_print -%>

<%= l.print\_label -%>

<% lpc = lpc + cllc -%>
<% if lpc > @lines\_per\_col -%>

<% lpc = 0 -%>
<% col = col + 1 -%>
<% if col > @columns\_max -%>

<% col = 1 -%>
<% end -%>
<% end -%>
<% end -%>

<% end -%>
