# Supplementary material for: Utilizing Descriptive Statements from the Biodiversity Heritage Library to Expand the Hymenoptera Anatomy Ontology
Source: PLoS One. 2013 Feb 18;8(2):e55674. doi: 10.1371/journal.pone.0055674 (PMC3575469; doi:10.1371/journal.pone.0055674)
Supplement: S1 Supplementary Material — The most recent version of mx code, including the Proofer tool, is available through SourceForge (http://purl.oclc.org/NET/mx-database). The specific version of mx used during analysis is archived on SourceForge and in this combined file. (ZIP) [file pone.0055674.s001.zip › ref_edge/app/views/layouts/application.html.erb]

MX - <%= self.controller.controller\_name.pluralize.gsub("\_", " ") -%>
<%= javascript\_src\_tag "svg\_web/svg.js", "data-path" => "/javascripts/svg\_web" -%>
<%= javascript\_include\_tag 'mx\_svgweb' -%>
<%= stylesheet\_link\_tag "standard" -%>
<%= class\_css -%>
<%= javascript\_include\_tag :defaults, :include\_google\_js => true, :versions => {:scriptaculous => "1.8.1", :prototype => "1.6.1"} -%>
<%= javascript\_include\_tag "accordion" if needs\_accordion -%>
<%= render(:partial => '/shared/colorPicker\_header') if needs\_colorPicker -%>
<%= render(:partial => '/shared/gmaps\_header') if needs\_gmaps -%>


<% if session && session[:person] -%>

Logged in as <%= session[:person].login -%>
(<%= link\_to 'logout', :controller => '/account', :action => :logout -%> |
<%= link\_to "my preferences", :action => 'preferences', :controller => 'person' -%> |
<%= wiki\_help\_link -%> |
<%= link\_to('SF', 'https://sourceforge.net/tracker/?group\_id=175927', :target => "\_blank") -%>)

<% end -%>
<% if @page\_title -%>

<%= @page\_title -%>

<% elsif @proj -%>

<%= @proj.name -%> ( <%= link\_to "settings", :controller => 'proj', :action => 'show', :id => @proj.id -%> | <%= link\_to "change projects", :controller => '/proj', :action => 'list' -%> | <%= link\_to "my data", :controller => '/proj', :action => 'my\_data' -%> )

<% end -%>
<% if @proj && @proj.id -%>
 
<%= menu\_tabs("main") -%>
<% else %>
<%= content\_tag('p') if !@page\_title -%>
<% end %>

<%= flash[:notice] -%>

<%# on all the time for AJAX %>
<% unless no\_subnav.include?(self.controller.controller\_name) -%>

<%= render(:partial => 'shared/class\_navigator') unless no\_sidenav.include?(self.controller.controller\_name) -%>

<%= menu\_tabs("subnav") -%>

<% end -%>
<%= yield -%>
