# Supplementary material for: Utilizing Descriptive Statements from the Biodiversity Heritage Library to Expand the Hymenoptera Anatomy Ontology
Source: PLoS One. 2013 Feb 18;8(2):e55674. doi: 10.1371/journal.pone.0055674 (PMC3575469; doi:10.1371/journal.pone.0055674)
Supplement: S1 Supplementary Material — The most recent version of mx code, including the Proofer tool, is available through SourceForge (http://purl.oclc.org/NET/mx-database). The specific version of mx used during analysis is archived on SourceForge and in this combined file. (ZIP) [file pone.0055674.s001.zip › ref_edge/app/views/layouts/stub.html.erb]

MX - <%= self.controller.controller\_name.pluralize.gsub("\_", " ") -%>
<%= stylesheet\_link\_tag "standard" -%>
<%= class\_css -%>
<%= javascript\_src\_tag "svg\_web/svg.js", "data-path" => "/javascripts/svg\_web" -%>
<%= javascript\_include\_tag 'mx\_svgweb' -%>
<%= javascript\_include\_tag :defaults, :include\_google\_js => true, :versions => {:scriptaculous => "1.8.1", :prototype => "1.6.1"} -%>
<%= javascript\_include\_tag "inputdraw" -%>
<%= javascript\_include\_tag "accordion" if needs\_accordion -%>
<%= render(:partial => '/shared/colorPicker\_header') if needs\_colorPicker -%>
<%= render(:partial => '/shared/gmaps\_header') if needs\_gmaps -%>


THIS LAYOUT IS A STUB - This content will be displayed within the layout placed in *public/site/your\_site/layout.html.erb*.

<%= yield -%>
