# Supplementary material for: Utilizing Descriptive Statements from the Biodiversity Heritage Library to Expand the Hymenoptera Anatomy Ontology
Source: PLoS One. 2013 Feb 18;8(2):e55674. doi: 10.1371/journal.pone.0055674 (PMC3575469; doi:10.1371/journal.pone.0055674)
Supplement: S1 Supplementary Material — The most recent version of mx code, including the Proofer tool, is available through SourceForge (http://purl.oclc.org/NET/mx-database). The specific version of mx used during analysis is archived on SourceForge and in this combined file. (ZIP) [file pone.0055674.s001.zip › ref_edge/app/views/mx/show_ascii.html.erb]

 matrix: <%=h @mx.name %> (id: <%= @mx.id %>) generated by mx on <%= Time.now %> 


matrix: <%=h @mx.name %> (id: <%= @mx.id %>) generated by mx on <%= Time.now %>

<% @chrs.in\_groups\_of( Person.find($person\_id).pref\_mx\_display\_width, false) do |int| %>

|  |<% int.each do |chr| -%> <%= chr.short\_name -%> |<% end -%>
| --- | --- |
<% i = 0 -%>
<% @otus.each do |otu| -%>|"> <%= otu.display\_name(:type => :matrix\_name) -%> |<% int.each do |chr| -%> <% @cs = ''; @cstxt = ''; -%> <% @codings\_mx[chr.id][otu.id].each do \|c\| -%> <% @cs += c.chr\_state\_state -%> <% end -%> <% if @cs.size > 0 -%> <%= @cs -%> <% else -%> — <% end -%> |<% end -%>
<% i == 0 ? i = 1 : i = 0 -%>
<% end -%>

  
<% end %>
