# Supplementary material for: Utilizing Descriptive Statements from the Biodiversity Heritage Library to Expand the Hymenoptera Anatomy Ontology
Source: PLoS One. 2013 Feb 18;8(2):e55674. doi: 10.1371/journal.pone.0055674 (PMC3575469; doi:10.1371/journal.pone.0055674)
Supplement: S1 Supplementary Material — The most recent version of mx code, including the Proofer tool, is available through SourceForge (http://purl.oclc.org/NET/mx-database). The specific version of mx used during analysis is archived on SourceForge and in this combined file. (ZIP) [file pone.0055674.s001.zip › ref_edge/app/views/ontology/test3.html.erb]

 
<%= javascript\_include\_tag "protovis-r3.0" -%>
<%= javascript\_include\_tag :defaults, :include\_google\_js => true, :versions => {:scriptaculous => "1.8.1", :prototype => "1.6.1"} -%>

<%= render :partial => '/ontology/visualize/protovis\_treemap' -%>
