# Supplementary material for: Utilizing Descriptive Statements from the Biodiversity Heritage Library to Expand the Hymenoptera Anatomy Ontology
Source: PLoS One. 2013 Feb 18;8(2):e55674. doi: 10.1371/journal.pone.0055674 (PMC3575469; doi:10.1371/journal.pone.0055674)
Supplement: S1 Supplementary Material — The most recent version of mx code, including the Proofer tool, is available through SourceForge (http://purl.oclc.org/NET/mx-database). The specific version of mx used during analysis is archived on SourceForge and in this combined file. (ZIP) [file pone.0055674.s001.zip › ref_edge/app/views/ontology/visualize/protovis_sunburst.html.erb]

Visualize
<%= javascript\_include\_tag :defaults, :include\_google\_js => true, :versions => {:scriptaculous => "1.8.1", :prototype => "1.6.1"} -%>
<%= javascript\_include\_tag "protovis-r3.0" -%>


|  |  |
| --- | --- |
| parents:  <%= @tree\_root.parents(:relationship\_type => @isa.id).collect{\|o\| content\_tag(:ol, link\_to(o.part2.display\_name, :action => :protovis\_sunburst, :id => o.part2.id))}-%>  <%= @tree\_root.display\_name -%> <% form\_remote\_tag :url => {:action => :\_set\_visusizliation\_preference, :id => @tree\_root.id}, :loading => "Element.show('update\_spinner')", :complete => "Element.hide('update\_spinner')" do -%> visualize: <%= select\_tag :overlay, options\_for\_select(["icicle", "sunburst", "treemap"], session["#{$person\_id}\_ontology\_vis\_pref"]) -%> <%= submit\_tag "go" -%><% end -%> | <% if @tree\_root.tree\_child(:relationship\_type => @isa.id).size > 0 -%><%= render(:partial => '/ontology/visualize/protovis\_treemap') -%> <% end -%> |
