# Supplementary material for: Utilizing Descriptive Statements from the Biodiversity Heritage Library to Expand the Hymenoptera Anatomy Ontology
Source: PLoS One. 2013 Feb 18;8(2):e55674. doi: 10.1371/journal.pone.0055674 (PMC3575469; doi:10.1371/journal.pone.0055674)
Supplement: S1 Supplementary Material — The most recent version of mx code, including the Proofer tool, is available through SourceForge (http://purl.oclc.org/NET/mx-database). The specific version of mx used during analysis is archived on SourceForge and in this combined file. (ZIP) [file pone.0055674.s001.zip › ref_edge/app/views/pcr/_worksheet.html.erb]

PCR worksheet from mx


|  |  |  |
| --- | --- | --- |
| Date:  <%= Time.now.to\_s(:long) -%><%= ", by: #{@done\_by}" if !@done\_by.blank? -%> | PCR DATA FORM |  |
| Primers:  <%= "#{@fwd\_primer.display\_name} / #{@rev\_primer.display\_name}" -%> |  |  |
| Protocol: <%= @protocol.blank? ? ' *none provided* ' : @protocol.description -%> | |  |

|  | PCR #   mx pcr id | extract id   mx extract id | specimen  mx specimen id(s) | water | dNTP | Buffer | MgSO4 | Primer (F) | Primer [R] | Taq | Template | Other | Total vol |
| --- | --- | --- | --- | --- | --- | --- | --- | --- | --- | --- | --- | --- | --- |
<% @pcrs.each\_with\_index do |p,i| -%>| <%= i + 1 -%> | <%= p.id -%> | <%= p.extract\_id -%> | <%= p.extract.display\_source\_identifiers -%> | <%= format("%.3f",@water\_per\_single\_rxn) -%> | <%= @dntp -%> | <%= @buffer -%> | <%= @mg -%> | <%= @primers -%> | <%= @primers -%> | <%= @taq -%> | <%= @templ -%> | <%= @other -%> | <%= @rxn\_vol -%> |
<% end -%>| - control | - | - | - | <%= format("%.3f",@water\_per\_single\_rxn) -%> | <%= @dntp -%> | <%= @buffer -%> | <%= @mg -%> | <%= @primers -%> | <%= @primers -%> | <%= @taq -%> | <%= @templ -%> | <%= @other -%> | <%= @rxn\_vol -%> |

|  |  |  |
| --- | --- | --- |
| **Totals for mix** (rows + 1) \|; Water \| <%= format("%.3f",@t\_water) -%>; dNTP \| <%= @t\_dntp -%>; Buffer \| <%= @t\_buffer -%>; MgSO4 \| <%= @t\_mg -%>; Primer [F] \| <%= @t\_primers -%>; Primer [R] \| <%= @t\_primers -%>; Taq \| <%= @t\_taq -%>; other \| <%= @t\_other -%>; Total \| <%= @t\_water + @t\_dntp + @t\_buffer + @t\_mg + @t\_primers \* 2 + @t\_taq + @t\_other -%> | Gel photo: | Misc notes <%= @notes -%> |
