# Supplementary material for: Utilizing Descriptive Statements from the Biodiversity Heritage Library to Expand the Hymenoptera Anatomy Ontology
Source: PLoS One. 2013 Feb 18;8(2):e55674. doi: 10.1371/journal.pone.0055674 (PMC3575469; doi:10.1371/journal.pone.0055674)
Supplement: S1 Supplementary Material — The most recent version of mx code, including the Proofer tool, is available through SourceForge (http://purl.oclc.org/NET/mx-database). The specific version of mx used during analysis is archived on SourceForge and in this combined file. (ZIP) [file pone.0055674.s001.zip › ref_edge/public/_doc/index.html]

Rails: Welcome on board


# Congratulations, you've put Ruby on Rails!

**Before you move on**, verify that the following conditions have been met:

1. The log directory and the empty log files must be writable to the web server (`chmod -R 666 log/*`).- The shebang line in the public/dispatch\* files must reference your Ruby installation.   
     You might need to change it to `#!/usr/bin/env ruby` or point directly at the installation.
   - Rails on Apache needs to have the cgi handler and mod\_rewrite enabled.   
     Somewhere in your httpd.conf, you should have:  
     `AddHandler cgi-script .cgi`  
     `LoadModule rewrite_module libexec/httpd/mod_rewrite.so`  
     `AddModule mod_rewrite.c`

Take the following steps to get started:

1. Create empty development and test databases for your application.  
   Recommendation: Use \*\_development and \*\_test names, such as basecamp\_development and basecamp\_test  
   Warning: Don't point your test database at your development database, it'll destroy the latter on test runs!- Edit config/database.yml with your database settings.- Create controllers and models using the generator in `script/generate`   
       Help: Run the generator with no arguments for documentation- See all the tests run by running `rake`.- Develop your Rails application!- Setup Apache with FastCGI (and Ruby bindings), if you need better performance

Having problems getting up and running? First try debugging it yourself by looking at the log files.   
Then try the friendly Rails community on the web or on IRC
(FreeNode#rubyonrails).
